# Quantifying human performance in chess

**DOI:** 10.1038/s41598-023-27735-9

**Published:** 2023-02-06

**Authors:** Sandeep Chowdhary, Iacopo Iacopini, Federico Battiston

**Affiliations:** Department of Network and Data Science, Central European University, 1100 Vienna, Austria

**Keywords:** Complex networks, Applied physics

## Abstract

From sports to science, the recent availability of large-scale data has allowed to gain insights on the drivers of human innovation and success in a variety of domains. Here we quantify human performance in the popular game of chess by leveraging a very large dataset comprising of over 120 million games between almost 1 million players. We find that individuals encounter hot streaks of repeated success, longer for beginners than for expert players, and even longer cold streaks of unsatisfying performance. Skilled players can be distinguished from the others based on their gaming behaviour. Differences appear from the very first moves of the game, with experts tending to specialize and repeat the same openings while beginners explore and diversify more. However, experts experience a broader response repertoire, and display a deeper understanding of different variations within the same line. Over time, the opening diversity of a player tends to decrease, hinting at the development of individual playing styles. Nevertheless, we find that players are often not able to recognize their most successful openings. Overall, our work contributes to quantifying human performance in competitive settings, providing a first large-scale quantitative analysis of individual careers in chess, helping unveil the determinants separating elite from beginner performance.

## Introduction

Countless individual careers shoulder the forward momentum in the sciences^1–5^, arts^6–10^ and sports^11–14^. Indeed, the recent availability of large-scale datasets is nowadays providing an unprecedented opportunity to study the drivers of human performance in all such different domains. In science, for example, Google Scholar allows to track individual careers of academics, where individual performances can be quantified via their impact, i.e. the attention received from the research community in form of citations. In areas like arts, despite the definition of *quality* being somehow elusive, new data has recently shed light on the role of early career exhibitions in reputed venues in the eventual success of the artist^7^.

Data-driven investigations of individual activity are a pillar of sports performance. Who can forget the success story described in the book *moneyball* for baseball^15^? The day Sandy Alderson realized that on-field strategies and player evaluations were better conducted by based on statistical data—than by the collective wisdom of old baseball men^15^, the game—as we knew it—changed. In tennis, network techniques suggested the identification of Jimmy Connors as the best player of the past^16^, a difficult task which requires arbitrary external criteria when comparing across eras. All in all, sport analytics are now commonplace in most major sports, providing clues for individual and team performance to boost success rates^17,18^. Interestingly, while sports have benefited from scientific methods, they have in turn become a frontier to develop new scientific tools to investigate success, innovation and learning, as one of the primary domains where growth and success are measurable in a data-driven fashion.

In this work we focus on individual careers in the competitive sport of chess. Moreover, chess is a highly intellectual activity which shares similarities to science. Thus, it is often located amid the two domains, a game where players use simple rules resulting in highly complex plays, often developing different personal styles able to influence long-term success in the game. Besides, the volume of online chess games freely available for analyses (several billions), makes chess a perfect candidate for testing hypothesis involving human performance in competitive settings. So far chess has predominantly been looked at at the level of single games. For example, past research focused on the role of memory in games^19^ and showed that opening popularity follows the well-known Zipf’s law^20^. However, these analyses did not use individual player-level data, treating games from different players on equal footing^19,20^, or focused on a small number of players^21^. Indeed, little attention has been devoted to individual careers and their evolution. In particular we ask—*what separates skilled players from the rest?* Earlier studies found that the answer is not intelligence^22^, and the role of deliberate practice remains heavily debated^23–25^.

Here we perform a comprehensive large-scale analysis of the habits of skilled and less skilled individual players over time, providing an anatomy of human performance in the popular game of chess. We characterize players’ careers in terms of hot-streaks, diversity and specialization in the opening sequences of their games, and analyze their diversity as a function of career stage. We find evidence for the presence of both hot and cold streak phenomena, revealing a surprising tendency for beginners to have longer hot-streaks as compared to expert players. By sequencing the opening moves of players at different skill levels, we show that beginners start with more diverse set of first moves, while advanced players and experts rarely start their games differently when playing as white. Yet, expert players display a broader response repertoire, showing the ability to surprise their opponent with a greater variety of responses. Moreover, when accounting for different variations of the openings, experts show a deeper knowledge of different variations within the same line, hinting at a deeper understanding of the game. Lastly, analyzing behaviour in time, we find that players explore more during the beginning of their careers, but tend to specialize using and exploiting only fewer openings at later career stages. Overall, our large-scale characterization of individual gaming behavior supports chess as a suitable laboratory to quantitatively investigate individual careers and human performance, demonstrating simple differences in playing habits and behaviours of beginners and experts.

## Results

**Figure 1:**
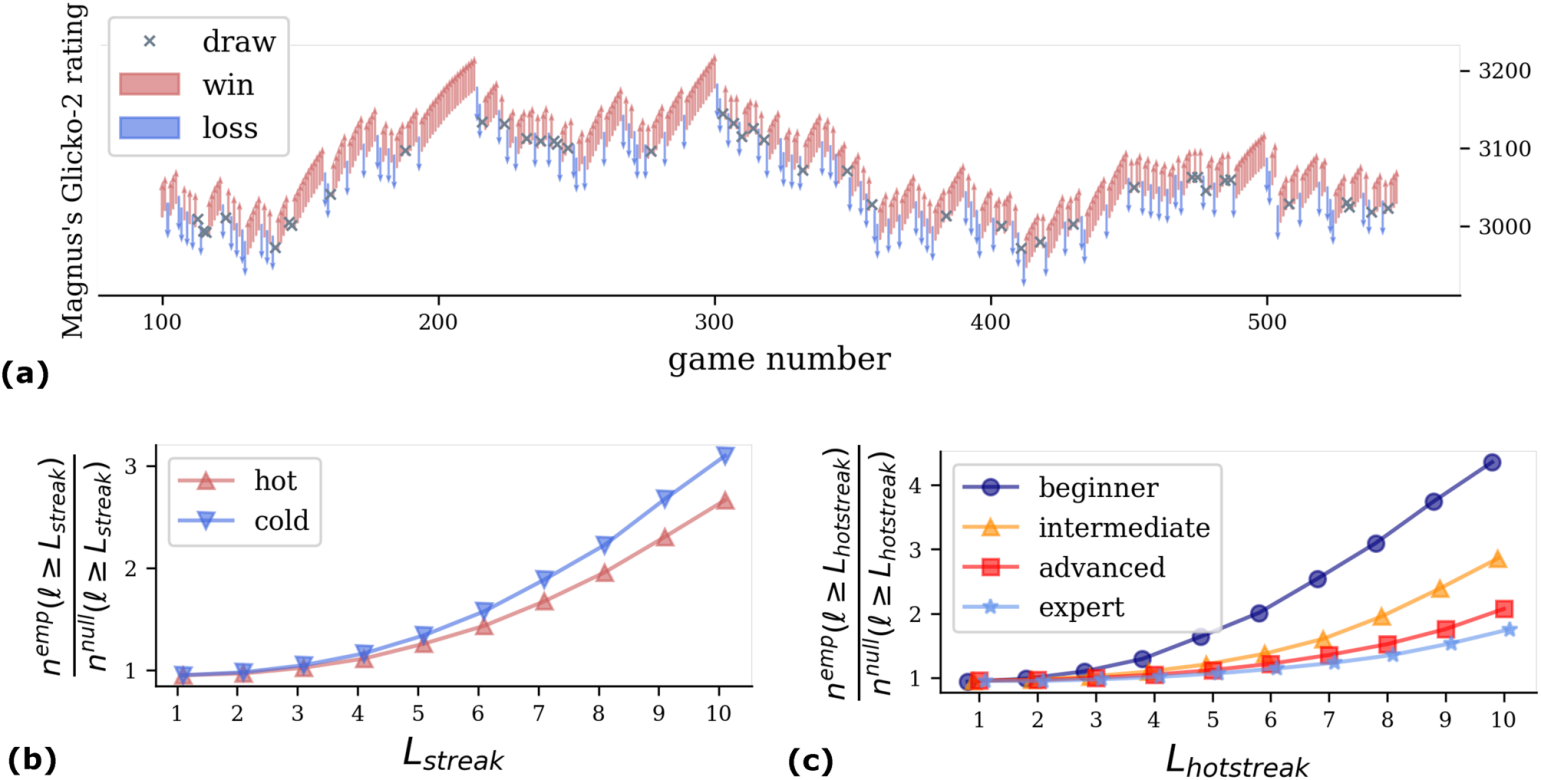
Hot and cold streaks in chess careers. Visualization of the career of the Grandmaster (GM) Magnus Carlsen. Wins and losses drive the *Glicko-2* rating up or down. (**a**) Relative number of hot streaks (red) and cold-streak (blue) of length $\ell \ge L_{streak}$ as a function of $L_{streak}$ calculated for each player. Results are averaged over all players. Losses tend to be more clustered than victories as individual cold streaks are on average longer than hot streaks. (**b**) Relative number of hot streaks of length $\ell \ge L_{hotstreak}$ as a function of $L_{hotstreak}$, averaged over the players in each skill categories separately (i.e. beginner, intermediate, advanced, expert). Weaker players have longer hot streaks than more expert ones.

In this work, we rely on large-scale data extracted from *lichess.org*, a popular open-source Internet chess server, consisting of 123 million games between 0.98 million players (see “Methods”). In the *lichess* dataset, each player’s career can be tracked over time, with detailed information on each of the played games, i.e. moves, opening, win/loss, and its skill level. This is quantified by the *Glicko-2* rating (see “Methods” for a detailed discussion of different ways to measure skill in chess), which measures the level of past performance of the player, it increases when a player beats an opponent and decreases upon a loss. As an illustrative example, in Fig. 1a, we show the career of Grandmaster (GM) Magnus Carlsen on *lichess.org*, indicating his *Glicko-2 rating* in each game and the game outcome: win, loss or draw.

Figure 1a seems to suggest that for *GM Carlsen* wins and losses tend to be clustered together. Indeed, prior works tracking wins and losses in sports hotly debate the existence of hot-streaks^26,27^, a phenomenon that has also been found to be ubiquitous in artistic and scientific careers^6,28^.

To quantitatively check for the existence of such phenomena in all chess careers, we calculate the length of hot (series of wins) and cold (series of losses) streaks for each player in the dataset, and compare them with lengths expected in a null model for each player which shuffles the temporal order of the player’s games, thus washing out temporal correlations in game outcomes (see “Methods” for details). In Fig. 1b, we show the resulting curves, properly normalised with the null model. We find the existence of statistically significant hot streaks, possibly associated with confidence spillovers from previous victories (in Sec. S6 we further confirm the presence of hot-streak using autocorrelation analyses^29,30^). Such hotstreaks could also occur simply due to consecutively facing weak opponents or large time-gaps between consecutive games. Controlling for skill, we find that the length of hot-streak and the average rating-advantage over the opponent in the streak are slightly correlated ($\rho _{speraman}\sim 0.28$ see Sec. S4), explaining only partially the observed behavior. For timegaps, we found a significant but very weak correlation ($\rho _{speraman}\sim 0.05$, see Sec. S5), hinting that timegaps between games do not play a major role in breaking hot-streaks. Our results are not affected by repeated matches against the same opponent. Indeed only $1.6\%$ player pairs play more than 5 games with each other, with a very large majority of opponents only meeting once (see Sec. S8). Long streaks of chess wins are reminiscent of players entering the so-called *zone*, a state of focus where peak performance is possible^31,32^.

Interestingly, cold streaks are also observed, typically longer than hot ones, indicating that times of poor performance tend to last more than periods of intense success. In physical sports, loosing streaks are often found to be the effect of an injury. Here, we speculate that similar phenomena might be in place even in chess, possibly due to lack of confidence, loss of focus and similar decrease in mind fitness more than purely athletic shape.

We can refine such analysis by further separating players by skill (Glicko-2 rating). Categorizing players into 4 categories—*beginner, intermediate, advanced, expert*  (see “Methods”). We find that weaker players experience comparatively longer hot streaks than stronger players (Fig. 1c). A reason for this could be that confidence spillovers from last victory may have greater impact on future outcomes at a lower skill levels.

Another possible driver of the observed disparity in hot streaks across beginners and experts can reside in how experts diversify their moves. In competitive sports, some players diversify their techniques while others may specialize. Strategy diversification might make players harder to predict, thus enabling them to surprise their opponents. By contrast, specialization, e.g., deeper knowledge of certain opening positions, may allow players to exploit opponents navigating familiar situations. Indeed, such an exploitation–exploration (specialization–diversification) dichotomy is a common mechanism governing the dynamics of many diverse self-organized and adaptive systems^33–36^.

**Figure 2:**
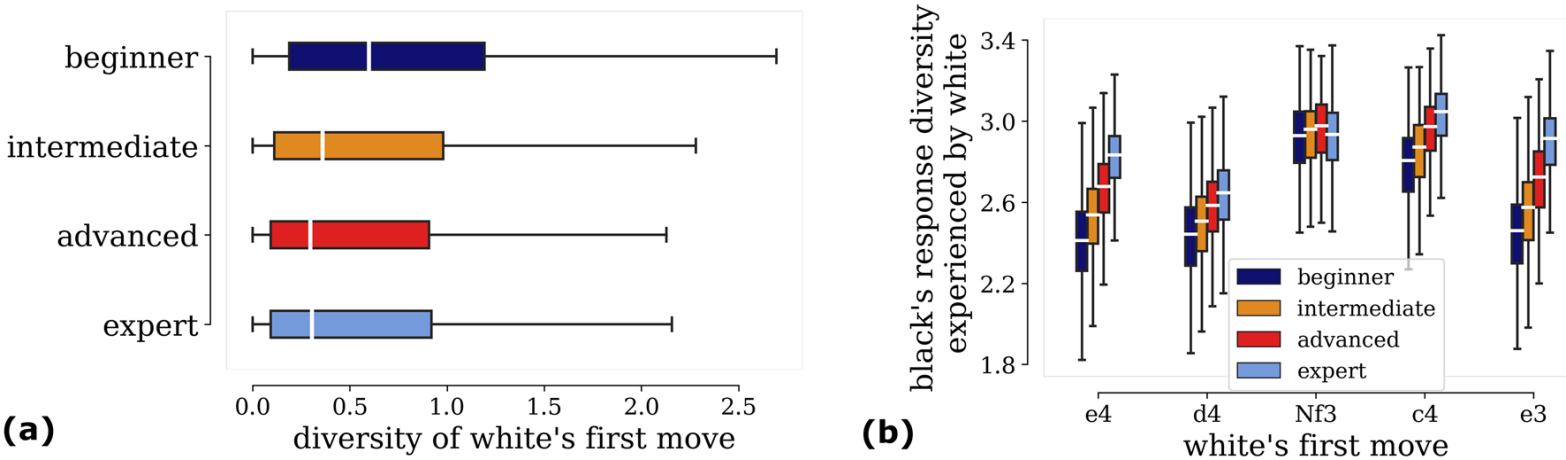
Diversity and specialization in the first move and black’s response. (**a**) Boxplots showing diversity (entropy) of *first move* by a player as white, calculated over all players individually and aggregated into the 4 different skill levels. Weak players start games with diverse collection of first move as white when compared to stronger players. (**b**) Boxplots showing diversity of black’s response experienced by white player, for each of white’s top 5 most played first moves—*e*4, *d*4, *Nf*3, *c*4 and *e*3 (in descending order of popularity). As white, weakest players encounter lowest diversity in responses captured by low response entropy—for all of white’s most played opening moves, except Nf3.

In chess—and sports in general—the balance of this trade-off may depend on skills. We thus investigate the extent to which skill level influences the approach to the game. In particular, we study the diversity in the player’s arsenal of game openings across different Glicko-2 ratings (Fig. 2). We calculate the Shannon entropy of the distribution (see “Methods”) of first move as white for each player and report the results in Fig. 2a. We find that beginners tend to open games with a diverse collection of first moves (as white) when compared to stronger players. Thus, our analysis captures beginners exploring a wider variety of first moves than experts, who instead are likely to begin with a typical move. At a first glance, this result might seem surprising, as skilled players are supposed to have better knowledge of opening theory. Yet, this may be linked to the ability of more skilled players to easily transpose into different opening variations in the following moves. Better awareness of transposition theory among experts may allow them to reach many different openings from the same starting move, thus potentially eliminating the need to diversify in the first move itself.

So, overall, do experts specialize at the cost of diversity? To investigate further, we ask—how does skill level determine response diversity (as black)? For the top 5 white moves observed—*e*4, *d*4, *Nf*3, *c*4 and *e*3, we group the games of each player based on these moves and calculate the response diversity of the black to the white player. Results are shown in the different boxplots of Fig. 2b. Surprisingly, we observe a contrasting result. As white, beginners encounter the lowest diversity in black responses. This is captured by the low response entropy for all 5 of white’s most played opening moves. Hence, beginners lack experience to the plethora of possible responses, which perhaps leaves gaps in their game.

Lastly, we point out that this increase in the diversity of responses at higher skill levels, might be what prevents players from increasing their Glicko-2 rating, as the potential to be surprised by your opponent keeps increasing as one climbs the skill ladder.

**Figure 3:**
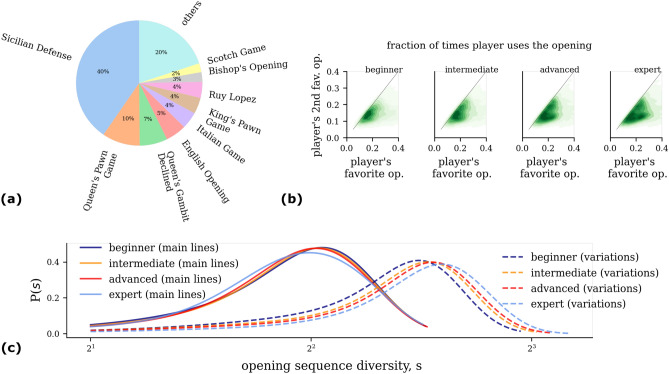
Diversity and specialization in the opening sequence of moves is governed by skill level. (**a**) Top 9 favorite openings among all players. (**b**) Fraction of times players use their top-2 favourite openings. Different panels correspond to different skill levels. Density plots are used for better visualization. Expert players play more often their favorite opening sequence as compared to beginners. (**c**) Distribution of diversity (entropy) of openings calculated for players of four different skill levels. Main lines and the variations are respectively depicted as solid and dashed curves.

From the first move onward, players enter into established chess theory, where the many top variations of opening moves are well-explored. The next natural question to ask at this point is—How do players diversify beyond the first move as player move into opening theory? The beginning usually plays out like a well-choreographed dance, evolving in already classified opening sequences with standard names such as “Sicilian Defense”, “Queen’s Pawn Game”, and so on. In Fig. 3a, we show the top 9 openings used by players on *lichess.com*. Focusing on such opening sequences, we explore the specialization players achieve in the opening sequence. Results are shown in Fig. 3b, where we define the “favorite opening” of a player as the most used one, assuming it is played at least 100 times.

Interestingly, the majority of players end up in their favorite openings only around  10–$30 \%$ of the time. Furthermore, we find that expert players start with their favorite opening significantly more times than their second favourite. This is marked by the distribution falling below the diagonal line. Contrarily, beginners lie much closer to the diagonal, indicating that their favorite opening is played comparably to the runner up, thus pointing out a lack of opening specialization.

Further analyses reveal that expert playing behavior comes in a variety of shapes and sizes, i.e., there are players who specialize and players who flexibly switch openings (diversify). This is marked by the bi-modal nature of the distributions observed in Fig. 3b, column 4.

At the individual level, we find on average less diversity in opening selection (main lines) among experts, as shown in Fig. 3c. As mentioned earlier, the ability to arrive into known openings through *transposition*, i.e., different sequences of moves that players may use to reach the same final configuration, might be unique to expert players. Arriving into fewer openings may allow experts to use learned chess theory and use optimal moves from memory, saving crucial time and preventing build-up of mental fatigue during the game.

However, accounting for the many different  *variations* of the openings (see “Methods”), it is the experts instead who encounter the most diversity. This hints that experts like to enter into certain main openings—perhaps the ones they specialize in—which they follow-up by expanding their repertoire in the *variations* to surprise opponents and catch them off-guard, a strategy not unique to chess but key in many competitive sports. Furthermore, upon investigating temporal organization of openings (main lines) used by a player, we find that experts switch openings between consecutive games more often than beginners (see Supplementary Information, Sec. S3). Thus, experts encounter higher temporal diversity in openings.

**Figure 4:**
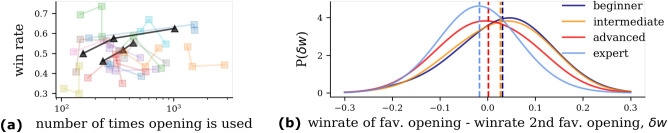
Sub-optimal opening encounters. (**a**) Winrate of top 3 openings of a player against opening frequency. Each connected curve corresponds to a player. We show 15 random players who play at least 100 games with each of their top 3 openings. Curves of players whose winrate increases monotonically with the frequency of the associated opening are depicted in black and are deemed *optimal*. (**b**) Distribution of difference $\delta w$ in winrate of associated to favourite and second favourite opening and winrate of their second favourite opening for the whole population of players. Different curves correspond to different skill levels. Dashed lines indicate mean values of the distributions. Stronger players encounter less optimal openings more often than weaker players.

At this point one might wonder—how much exactly does specialized knowledge of favorite openings aid in victory? A naive argument would suggest that players would tend to prefer those main lines that give them the best results. If this is the case, the favourite opening of each player—the one mostly used—would be the one that gives the best performance, that is the highest winrate. To investigate this, we calculate for each player the winrate of each of the player’s top-3 most played openings and plot it against the frequency of their use. Results are shown in Fig. 4a for a sample of the players. Surprisingly, there are players whose top used opening performs worse than their lesser used openings. Besides, optimal players (black curves)—those who play more often their better performing openings—are just a few.

To quantify this effect in the whole population, we calculate for each player the difference in the winrate of the most played opening and the second most played one, showing its distribution in Fig. 4b. Our analysis reveals that when expert players do encounter their favorite opening, their winrate is more likely to be lower than their second favourite opening, when compared to beginners. We note that players who do better in their second most played opening—as compared to their most played one—are experiencing sub-optimal opening encounters. Thus, we find that stronger players encounter sub-optimal openings more often than weaker players. In other sports, players are known to change their strategies over opponents and games so that specific habits are not exploited by opponents for strategic advantage. We speculate that such variations from optimal strategy might serve only a minor role in online play, where opponents are randomly selected from a large pool of millions of players. Thus, discovered sub-optimal encounters may be an opportunity for players to improve.

**Figure 5:**
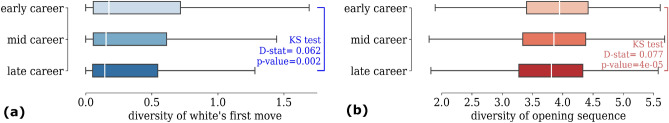
Diversity in opening with career stage. We show the diversity in the opening move (**a**) and opening sequence (**b**) of moves. In later parts of one’s career, diversity decreases, players prefer certain openings and specialize in them—playing them more often.

Lastly, we explore diversity as a function of different stages of players’ careers. Selecting players with at least 3000 games, we split them into 3 equal stages: early (0–1 k), mid (1–2 k) and late career (2–3 k). For each play, we compute opening diversity in the different career stages and report it in Fig. 5. For both the opening move (a) and the opening sequence (main lines) (b), we find that players explore more in the initial stages of their careers, becoming more specialized in later stages, perhaps exploiting the knowledge of certain openings they have learned.

## Discussion

Quantifying performance and unveiling the drivers of success is an ubiquitous pursuit in modern society, especially important in competitive settings, where skills, techniques, strategies, and achievements need to be compared. Indeed, in many sports, measuring and analysing performance has nowadays become a common practise^37,38^. In this work, we propose chess as a natural laboratory to investigate human behavior^39–42^. Differently from most other disciplines, chess has no stochastic component, hence performance can be more directly associated with skill—as quantified by Glicko-2 rating. Having this in mind, here we performed a data-driven investigation of almost 1 million individual careers carving their way to success in the online platform *lichess.org*. Looking at entire careers, we found the presence of hot and cold streaks. These are bursts of victories and losses, already observed in science and other domains^6,9,28^, which might be due to periods of particularly successful physical fitness, creativity or even confidence. Accounting for skill level, we noticed that beginners have a higher chance to experience a repeated sequence of wins, and that the typical length of a hot-streak is inherently related to the skill level of a player. Moreover, no matter individual ability, player performance is characterized by even longer period of repeated failure.

Even just looking at simple patterns in the openings—thus neglecting the full complexity of game sequences, we were able to characterise individual playing behavior across different career stages. In particular, expert players were shown to behave differently from the very first move of the game, displaying a lower diversity in openings. Looking at chess as a process of interactions and reactions, we focused on the black’s response to the white player’s moves, finding that experts encounter the highest diversity from black. However, after accounting for different variations within the openings we discovered that experts were more diverse instead, hinting at a deeper understanding of the complexity of the different variations within the same line. Such findings corroborates some very recent ideas on opening similarity and complexity independently presented in Ref.^43^, focusing on prediction of future openings and opening preparation, about which we recently became aware. Looking at individual careers over time, opening diversity was found to decreases at their later stages, pointing towards higher specialization as a player becomes more experienced. In addition, experts tend to play their favorite opening sequence much more than beginners, providing evidence for a tendency towards specialization. Nevertheless, counter-intuitively, we also found that players often do not have the ability to recognize their most successful opening, i.e. the one associated with the highest win-rate. Surprisingly, this is particularly true for more expert players, who have a higher chance of sub-optimal encounters in opening, possibly because of the depth of responses and variations within opening lines coming from a skilled opponent. Indeed, in general decision making^44–46^ and particularly in chess^41,47^, humans are known to work with heuristic approaches relying of intuition rather than searching for optimal solutions. The observed deviation from optimal strategies in our data might hence also hint at the existence of adaptive strategies with “fast and frugal” heuristics by players^48^.

The study we have presented has two main limitations. First, we kept our focus on openings only, which constitute one of the many phases of chess games. Nevertheless, this simple approach proved to be enough to reveal how experts differ from beginners in simple quantifiable ways. It also complements existing work on recall abilities of players for chess positions as a function of skill level^40^. A first natural extension in this direction would consist in analysing also other parts of the game, such as middle game and endings. A second limitation is that, when associating a skill level to a player, we inevitably considered Glicko-2 rating as a static, immutable measure. Instead, this rating systems is clearly in constant evolution throughout the career of a player. While including this dynamical aspect of ranking would surely add a missing aspect to the analysis, it is worth stressing that our measure is still a good proxy for skill level, as we have neglected the initial phase of the careers—associated to the steepest growth/change in Glicko-2 rating.

Taken together, our work represents a first step towards understanding the game mechanisms associated to performance in the careers of chess players. Future work might enrich this analysis by considering the complexity of chess games as a whole via considering the full sequences of moves instead of focusing on the important phases of the game only. Finally, and more broadly, it would be interesting to extend our approach to other ecosystems, investigating tensions between specialization and diversity in other contexts—from Go to tennis and boxing matches—where opening and response constitute a crucial part of the game.

## Methods

### Data

For our analysis, we use all games played on the online chess server *lichess.org* between 2013 and 2016. Such data covers 123 million games played between 0.98 million players. There are different games available to the players on the platform: bullet, blitz, and rapid. The analysis presented in this paper is restricted to Blitz games, which are fast and tactical but still allow for some strategy in the game overall unlike bullet games which last only 1 minute at most and littered with pre-moves. The most popular time controls for blitz are 5 mins and 3 mins. We specifically focused on this type of games since “speed” chess is played across all levels, from beginners to grandmasters.

### Measuring diversity

We measure the diversity of openings of a player by calculating the Shannon entropy^49^ of the distribution of frequency of opening moves or opening sequences (see Figs. 2, 3 respectively). Notice that for the analysis in Fig. 2b, we selected only games where the player starts as white.

### Null models for hot and cold streaks

To calculate the expected lengths of hot streaks in a player’s career, we build a null model where we reshuffle the temporal order of the player’s games, but preserving the total number of victories, losses and draws. Such shuffling of the order of games within each player’s career breaks the temporal correlations between game outcomes^6^.

Then, we compute the length of each hot and cold streak (sets of consecutive wins and loses) observed in this reshuffled sequence. The presence of hot- and cold-streak phenomena can be then investigated by comparing the number of hot and cold streaks of a given length $\ell$ in the actual careers with respect to these reshuffled sequences.

### Chess concepts

#### Openings

A chess opening is the initial stage of a chess game—a sequence of first few moves. It usually consists of established theory; the other phases are the middlegame and the endgame. All games can be associated with a unique main opening line, within which there can be many variations. Many opening sequences have standard names such as the Sicilian Defense, Ruy Lopez, Italian Game, Scotch Game etc.

#### Chess rating systems

We present a very short overview of different rating approaches developed for chess. Our analyses are based on the Glicko-2 rating systems.

*Elo system*, invented by Arpad Elo,is the most common rating system for chess. It is used by FIDE, other organizations and some Chess websites such as Internet Chess Club and chess24.com. Games are scheduled by matching together players of similar ratings.

*Glicko-1 system*^50^ is a more modern approach, invented by Mark Glickman as an improvement of the Elo system, which preserves the philosophy or the Elo rating approach while making it more accurate. In the Glicko system, a player’s rating not only changes due to game outcomes, but also from their “ratings deviation”, which measures the uncertainty in a rating due to both game outcomes and also from the passage of time when not playing. At the cost of being more mathematically complex, the Glicko rating system is known to have a better prediction accuracy than Elo, and it is a popular choice for new games and sports. The Glicko system has a initial rating starting at 1500. It is used by Chess.com, Free Internet Chess Server and other online chess servers.

*Glicko-2 system* is a refinement of the original Glicko system and is used by Lichess, Australian Chess Federation and other online websites. It achieves even better accuracy by controlling for volatility. Volatility measures the degree of expected fluctuation in a player’s rating—it is low when the player performs at a consistent level and high when a player has erratic performances (e.g., when the player has had exceptionally strong results after a period of stability) We show the Glicko-2 ratings of all players in our dataset in Supplementary Information (Sec. S1). The career lengths of players as a function of their skill is shown in Supplementary Information (Sec. S2). Initial ratings start at 1500. Here, we associated to each player its rating averaged in all their games (to account for common early fluctuations, for each player we do not consider their rating in the first 100 games). As shown, on average experts tend to play more games than beginners. We note that players of similar ratings are matched to compete. However, small rating differences among matched players might persist. In Sec. S7, we show that such small rating differences are partly associated with game outcomes.

#### Separating players by skill level

We separated the player into the 4 skill levels as follows. We first arranged the players in ascending order of their *Glicko-2* rating (average calculated over all their games). We then created Glicko-2 rating bins that divide players in 4 equally sized skill categories. Finally, we labelled these bins as—* beginner, intermediate, advanced, expert* respectively.

#### Opening variations

For a given chess opening there are multiple variations, as players can explore different moves after the main opening line is established. For example, the *Sicilian Defence* begins with the following moves 1. e4 c5. The *Sicilian Defence: Najdorf Variation* of Sicilian is 1.e4 c5 2.Nf3 d6 3.d4 cxd4 4.Nxd4 Nf6 5.Nc3 a6, while the *Sicilian Defence: Dragon Variation* is 1.e4 c5 2.Nf3 d6 3.d4 cxd4 4.Nxd4 Nf6 5.Nc3 g6.

## Supplementary Information

Supplementary Information.

## Data Availability

The data from lichess.org used in this work is openly accessible for download from https://database.lichess.org/.

## Code availability

The code used in this study is available at https://github.com/chowdhary-sandeep/lichess.git.
