# Author Correction: Quantifying human performance in chess

**DOI:** 10.1038/s41598-023-31622-8

**Published:** 2023-03-23

**Authors:** Sandeep Chowdhary, Iacopo Iacopini, Federico Battiston

**Affiliations:** Department of Network and Data Science, Central European University, 1100 Vienna, Austria

Correction to: *Scientific Reports* https://doi.org/10.1038/s41598-023-27735-9, Published online 06 February 2023

The original version of this Article contained an error in Figure 2b, where the labels of the X and Y axes were swapped.

The original Figure 2 and accompanying legend appear below.

**Figure 2:**
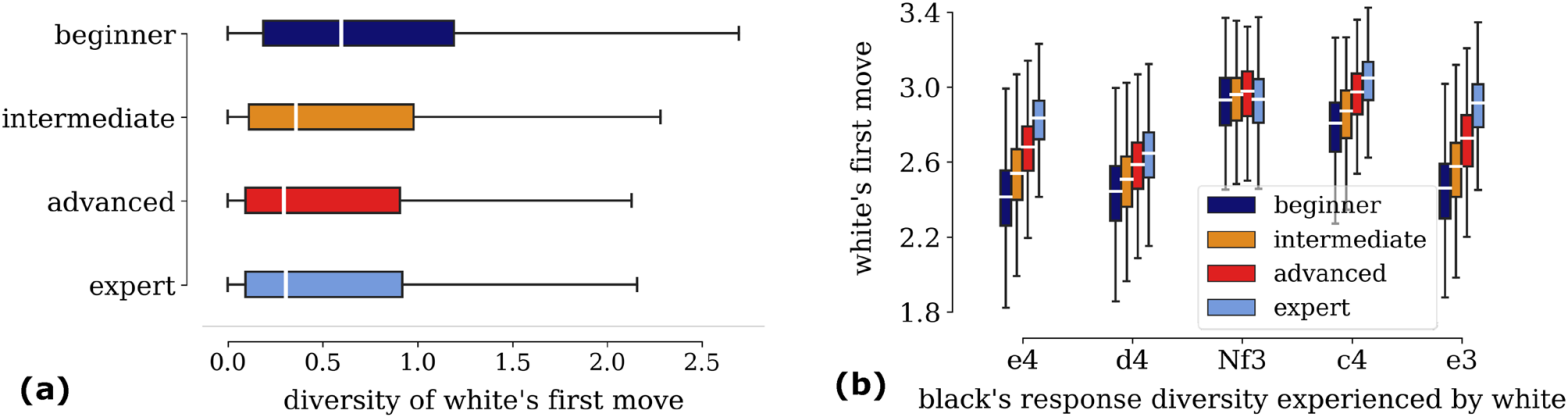
Diversity and specialization in the first move and black’s response. (**a**) Boxplots showing diversity (entropy) of *first move* by a player as white, calculated over all players individually and aggregated into the 4 different skill levels. Weak players start games with diverse collection of first move as white when compared to stronger players. (**b**) Boxplots showing diversity of black’s response experienced by white player, for each of white’s top 5 most played first moves—*e*4, *d*4, *Nf*3, *c*4 and *e*3 (in descending order of popularity). As white, weakest players encounter lowest diversity in responses captured by low response entropy—for all of white’s most played opening moves, except Nf3.

The original Article has been corrected.

