# Supplementary material for: Quantifying human performance in chess
Source: Sci Rep. 2023 Feb 6;13:2113. doi: 10.1038/s41598-023-27735-9 (PMC9902564; doi:10.1038/s41598-023-27735-9)
Supplement: Supplementary file 1 — Supplementary Information. [file 41598_2023_27735_MOESM1_ESM.pdf]

Supplementary Information for

Quantifying human performance in chess

S. Chowdhary, I. Iacopini, F. Battiston

*Corresponding author email: chowdhary_sandeep@phd.ceu.edu, battistonf@ceu.edu

S1 Glicko-2 rating distribution of the population

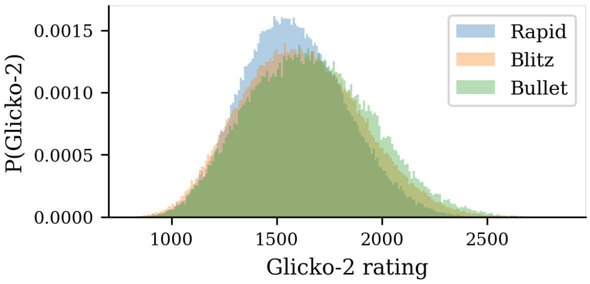

Figure S1. Distribution of Glicko-2 ratings averaged over the career of a player separately for the different time controls i.e. Bullet, Blitz and Rapid.

S2 Career length distributions vs skill level

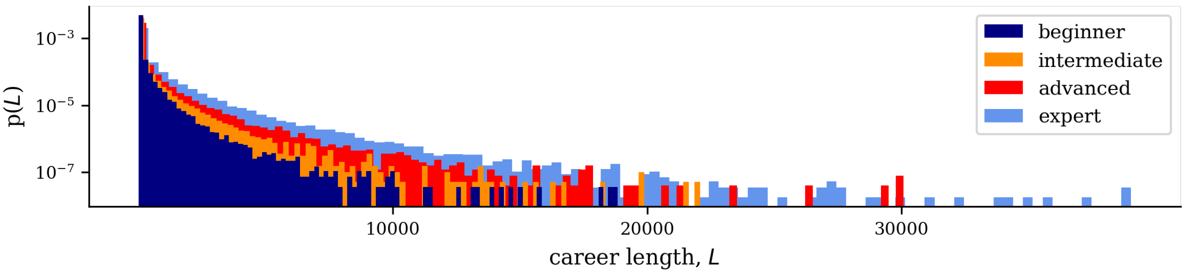

Figure S2. Distribution of the total number of games by a player for the 4 skill categories

S3 Opening switches in a career vs skill level

We calculate the number of opening switches between consecutive games for each player in the dataset, and compare them with the number expected in a reshuffled null model for each player. For the null model, we reshuffle the temporal order of the associated sequence of games, thus preserving the total number of victories, losses and draws.

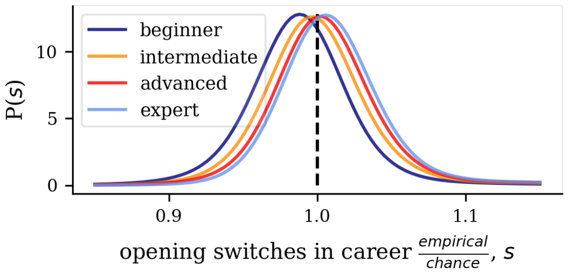

Figure S3. Distribution of the number of opening switches in a player's career properly normalised with the null model aggregated into 4 skill categories.

S4 Hot-streaks and rating advantage over opponent

Players are selected to play together if they have similar rating scores. However, small rating differences might still exist, and impact the length of hot streaks, which could be positively influenced by consecutively facing weaker opponents. To test this, in Fig. S4 (top) we plot the average rating advantage over the opponent in hot streaks as a function of their length. Results are averaged by considering all the games composing a given hot streak of a certain length, and over all hot streaks of that length. We find that longer hot streaks are associated to a higher average advantage over opponent, thus supporting the idea that weaker opponents might cause hot-streaks. We quantify this effect by performing a Spearman's correlation test between the length of hot streaks and the average rating-advantage over the opponents in the streak. We find a small correlation, $\rho \sim 0.28$, which is statistically significant ($p < 10^{-6}$), and which might partially explain the observed behavior. In Fig. S4 (bottom), we also compare the rating advantage over the opponent who broke the hot-streak with respect to the one of the opponent in the preceding game (the last one composing the hot-streak) as a function of hot-streak length. The relative advantage is negative, which implies that opponents who break a hot-streak are consistently stronger than opponents who got beaten during the hot-streak. However, we note that this result is somehow expected, as in the chess gaming platform analysed in our work a player who keeps winning consistently (in a hot streak) will likely be matched with higher-rated opponents in the next game.

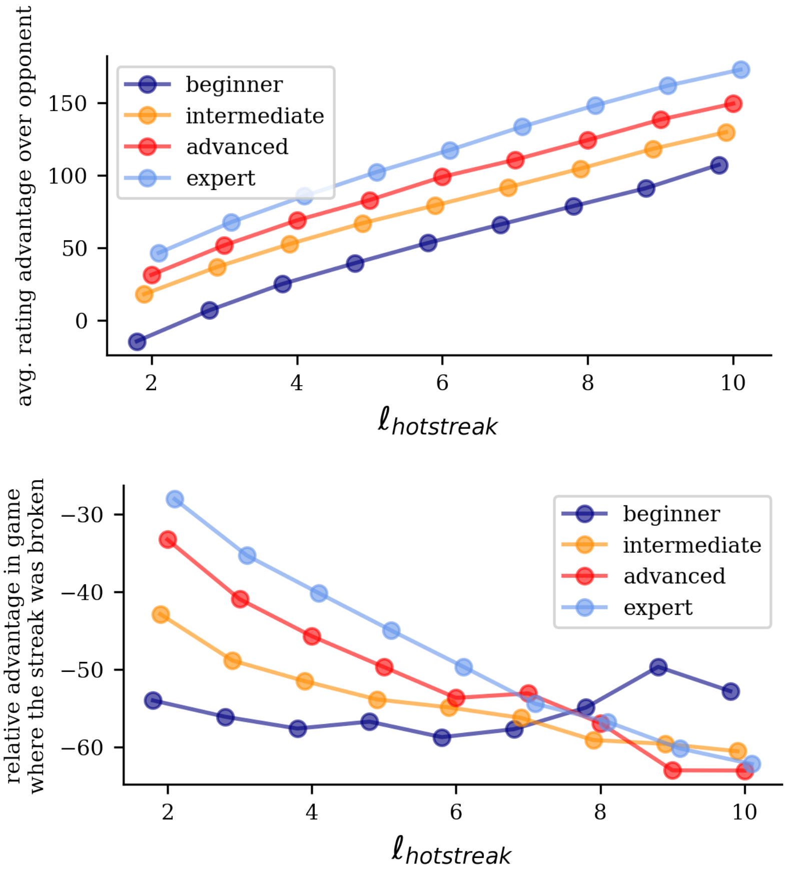

Figure S4. Average rating advantage over opponent during the hot-streak as a function of hot-streak length $\ell_{hotstreak}$ (top). Rating advantage in the streak ending game compa to the advantage in preceding game as a function of hot-streak length $\ell_{hotstreak}$ (bottom).

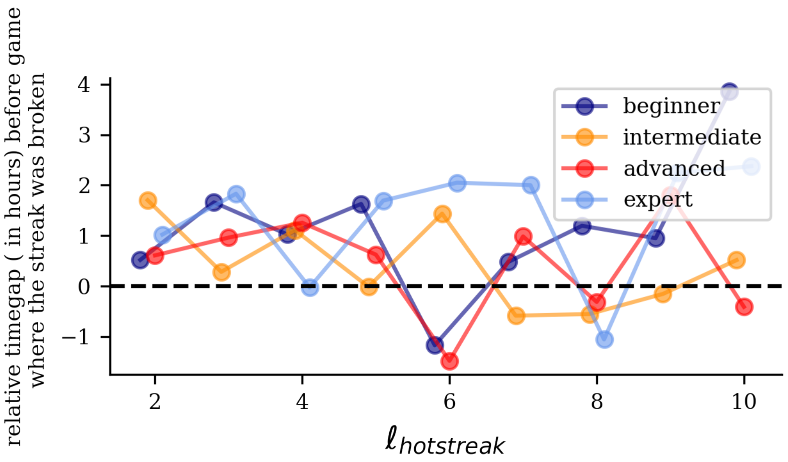

Figure S5. Relative timegap before streak-ending game– calculated as the difference of timegap before the streak ending game compa to the timegap in preceding game as a function of hot-streak length $\ell_{hotstreak}$.

S5 Hot-streaks and time-difference between successive games

We also investigated the effect of time-difference between games and possible breaking of hot-streaks due to time gaps. In Fig. S5 we plot the difference between the time-gap before the game where the hot-streak was broken and the time-gap before the preceding game as a function of hot-streak length. For short hot-streaks (lengths 2,3,4) we find a positive relative time-gap before the streak ending game, hinting that such hot streaks could also be disrupted by player taking a break before the next game. The overall trend remains unclear for longer streaks, which are the ones which are really associated with the so-called hot streak phenomenon. We quantify this effect by performing a Spearman's correlation test between the time-gaps before the streak breaking game and the length of the hot-streak. We find a significant but very weak correlation($\rho \sim 0.05$).

S6 Measuring hot-streaks effect via autocorrelation

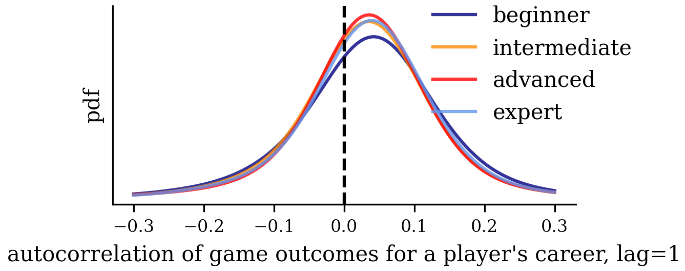

Figure S6. Distribution of autocorrelation in game outcomes for a player's career with lag 1 aggregated into 4 skill categories.

Our definition of hot-streaks is based on a hard constraint, where one loss ends a hot-streak. A more loose statistical way of quantifying hot-streak phenomena is via measuring autocorrelations. We compute auto-correlation with lag 1 among the outcomes and find it to be positive for all skill levels (Fig. S6). This finding further corroborates the presence of hot-streaks discussed in the main manuscript.

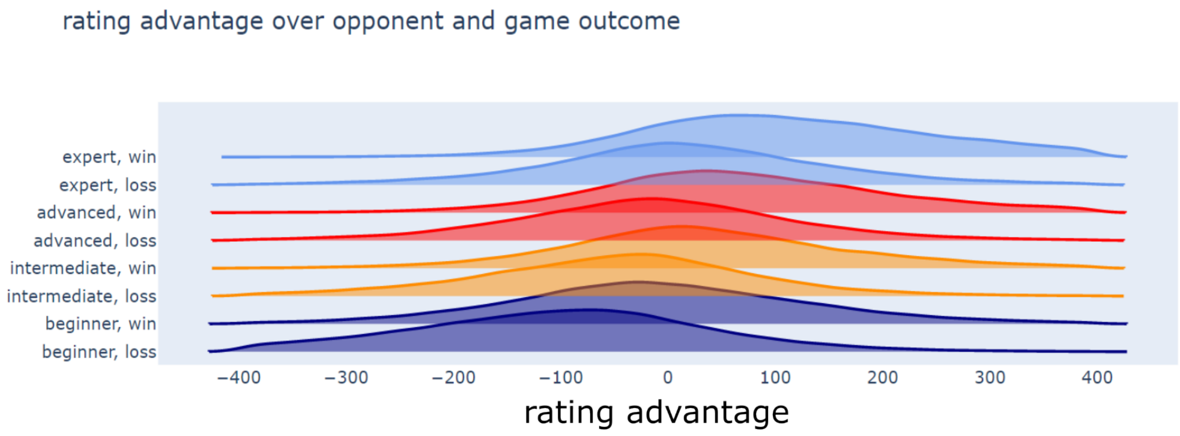

Figure S7. Distribution of rating advantage over opponent for games as a function of game outcome– win or loss– for the 4 skill categories.

S7 Rating advantage over opponent for games as a function of game outcome

To check how game outcomes are affected by small rating differences (the only ones possible for players who are matched to play against each other), we plot the distribution of advantage over opponent for games as a function of game outcome– win or loss (Fig. S7). We observe that such small positive rating differences are indeed associated with a higher chance to win a game.

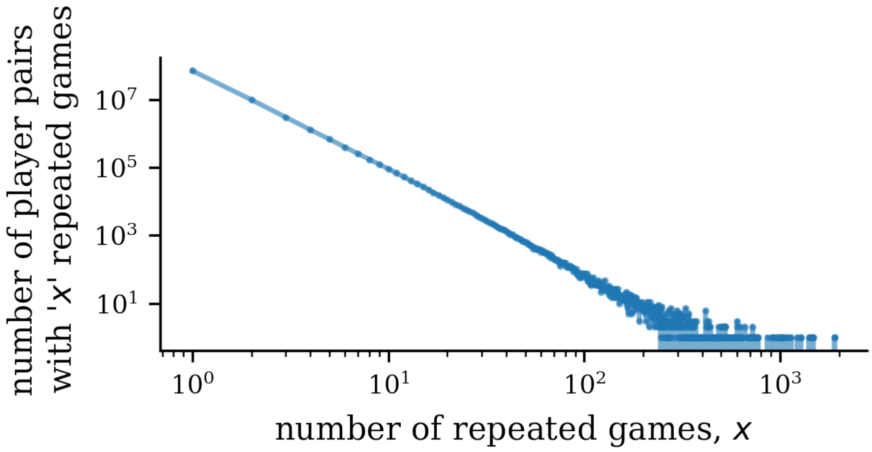

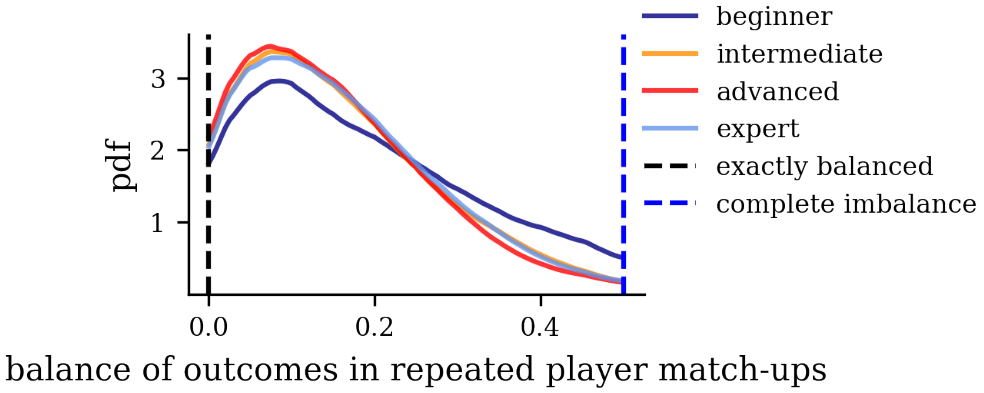

Figure S8. Distribution of number of repeated matches (top). Distribution of balance in outcomes in player match-ups (bottom) for the 4 skill categories. A match-up is balanced (=0) if the players had as many wins as losses, and completely imbalanced (=0.5) if one players always beats the other.

S8 Effect of repeated matches with the same opponent on hot-streak behaviour

In other sports, it is well known that players need to vary their strategies against an opponent, so that habits are not exploited for strategic advantage. However, such variation in strategy is not very relevant in online chess games, where repeated matches are rare. Fig. S8 (top) shows the frequency of repeated matching of players. We find that 81% of player matches never repeat, and only 1.6% player pairs play more than 5 games with each other. Thus, repeated games do not significantly affect our results.

In addition, to specifically check what happens when opponents are repeated, we investigate the balance of wins and losses in repeated match-ups among two players (with at least 20 games between them). In Fig. S8 (bottom), we show the balance of outcomes, computed as $|\frac{n_{wins}}{n_{games}} - 0.5|$. We find that while a majority of repeated match-ups lean towards balance (50-50), there exist some pairs which are highly imbalanced, with one player dominating and winning consistently, which might result in some hot-streaks. However, as stated earlier, these repeated games represent only a tiny fraction of all the games and will not affect significantly our results.
